# Sexual identities and reproductive orientations: Coming out as wanting (or not wanting) to have children

**DOI:** 10.1177/1363460720926967

**Published:** 2020-06-11

**Authors:** Robert Pralat

**Affiliations:** 2152 University of Cambridge, UK

**Keywords:** coming out, generation, identity, normativity, orientation, parenthood, reproduction, sexuality

## Abstract

In the context of growing visibility, recognition and acceptance of lesbian motherhood and gay fatherhood in countries such as Britain, it is important to ask how younger generations of sexual minorities approach the possibility of becoming a parent. Drawing on interviews with lesbians and gay men who do not have children but may have them in the future, I explore how people become aware that having children is an option. By attending to how this consciousness manifests in conversations and how conversations shape the consciousness, I illuminate specific dynamics that raising the topic of parenthood creates in intimate interactions. My data show that it is often unclear to men and women who form same-sex relationships whether they are socially expected to have children. I argue that this ambiguity requires a kind of ‘coming out’ through which feelings about parenthood are made explicit. Using the concept of coming out, I ask: What if we were to think of people in terms of their ‘reproductive orientations’ rather than sexual identities? I suggest that, similar to expressing sexual identities, articulating reproductive orientations involves aligning with particular life trajectories based on binary logic. However, with ambiguous expectations about parenthood, neither having children nor remaining childfree is explicitly normative. As such, unlike coming out as lesbian or gay, which transgresses norms surrounding sexuality, coming out as wanting or not wanting to have children challenges normativity itself. I reflect on how this ‘normative challenge’ makes it possible to imagine parenthood and ‘childfreedom’ as intimacies of equal value.

## Introduction

Since the early 2000s, there has been a rapid shift in Britain and other western countries towards greater public visibility, legal recognition and social acceptance of non-heterosexual or queer parenthood.^1^ Studies of lesbian mothers and gay fathers have documented experiences of the first generations of ‘intentional’ sexual-minority parents – people who have children after ‘coming out’ as lesbian or gay – sometimes describing them as ‘pioneers’ (Dunne, 2000; Ryan-Flood, 2005; Stacey, 2006). Queer kinship has provided exemplar ‘modern families’ (Gamson, 2015; Golombok, 2015; Tober, 2018) and, arguably, it has never been more evident that same-sex intimacy and parenthood are not mutually exclusive. One would expect that the expanded notion of the family has made it easier for new generations of non-heterosexual people to see parenthood as a future possibility. In this article, I ask: How is the apparent expansion of parenthood possibilities beyond heterosexuality experienced by potential beneficiaries of this cultural transformation? Focusing on lesbians and gay men in their 20s and early 30s, who were born in the 1980s, grew up in the 1990s and entered adulthood at the turn of the century, I examine how people become aware of the fact that having children in a same-sex relationship is an option. How does this consciousness manifest in conversations? And, conversely, how do conversations shape the consciousness?

Using data from a small-scale interview study, I explore what lesbians and gay men in Britain, who do not have children but may have them in the future, think about the possibility of becoming a parent and how they talk about it with their peers, including partners and friends. Through an analysis of how thinking about parenthood translates to talking about it, and vice versa, I show that, at a time of cultural change, it is often unclear to people who form same-sex relationships not only whether they are socially expected to have children but also what their queer peers are more likely to want. I argue that both sexual identities (how we think of ourselves in terms of sexual attraction) and what we can understand as ‘reproductive orientations’ (how we think of ourselves in terms of the willingness to have children) involve aligning with particular life trajectories which are based on binary logic. I suggest that expressing one’s parenting desire, or lack thereof, is a kind of ‘coming out’ through which feelings about parenthood are made explicit. However, in a context of ambiguous cultural expectations about parenthood, neither having children nor remaining childfree is explicitly normative. As such, unlike coming out as lesbian or gay, which transgresses norms surrounding sexuality, coming out as wanting (or not wanting) to have children challenges normativity itself. This ‘normative challenge’, I conclude, makes it possible to imagine parenthood and ‘childfreedom’ as intimacies of equal value.

Before presenting my findings and developing my argument, I situate this study within the social science scholarship on lesbian motherhood, gay fatherhood and same-sex intimacy. I sketch a conceptual framework for thinking about the relationship between reproduction, sexuality and identity, and explain what exploring the perspectives of sexual minorities who have not experienced parenthood but who might become parents in the future can add to our understanding of queer kinship in younger generations.

## Identity conflicts and turning points

Much of the academic literature on lesbian motherhood and gay fatherhood, especially the early scholarship on this topic from the USA, emphasises tensions between sexual and parental identities, often perceived as in conflict by lesbians and gay men themselves. As studies of lesbian mothers highlight, towards the end of the last century, lesbian motherhood was still considered a ‘contradiction in terms’ (Lewin, 1993: 1), an ‘oxymoron’ (Hequembourg and Farrell, 1999: 541). Early research on gay fatherhood similarly notes that ‘the term gay father may seem antithetical’ (Bozett, 1989: 138). More recent literature echoes these prior observations. For example, in a study of gay, lesbian and heterosexual adoptive parents in Britain, Jennings et al. (2014) report that many parents in same-sex couples reflected that, when they were younger, accepting their sexual identity meant simultaneously accepting childlessness. The authors note that this was especially the case for gay fathers and for older parents.

Indeed, gender and generation are two interacting factors that seem to influence the likelihood of experiencing an identity conflict. The idea of the mutual exclusiveness of the two identities, and of the necessity to redefine the meaning of one’s sexual identity when pursuing parenthood, is most salient in studies of older gay fathers from earlier generations (Lewin, 2009; Mallon, 2004). Authors often observe generational shifts within their own data. For example, Murphy, in his study of Australian and American gay men who became parents through surrogacy, reports that most men ‘initially accepted or acknowledged the notion that equated homosexuality with childlessness’ (Murphy, 2013: 1120) and, for those over 40 years of age, ‘coming out as gay meant almost certain childlessness’ (Murphy, 2013: 1111). Similarly, Berkowitz and Marsiglio, who studied both gay fathers and gay men without children in the USA, report that ‘many of the participants, especially those older than 35 years, viewed the coming-out process as synonymous with the realization that they will never be fathers’ (Berkowitz and Marsiglio, 2007: 372). These generational shifts are less explicit in studies of lesbian mothers, although, as Gabb observes in her recent reflections on lesbian motherhood over a generation, in the 1990s, the power of heteronormative ideology made it hard to ‘reconcile parental and sexual identities’, which shaped ‘the boundaries of what was imaginable’ for women in the realm of queer parenthood (Gabb, 2018: 1009).

Research on younger generations of sexual minorities evidences both a significant change in thinking about parenthood and how it continues to be shaped by gender. For instance, in a study of couple relationships in Britain, Gabb and Fink found that, for many queer couples, whether or not to have children was a decision ordinarily discussed and, for younger couples in particular, ‘it was the choices of “when” and “which way” to conceive that seemed to perplex them’ (Gabb and Fink, 2015: 102). Likewise, in a UK study of same-sex couples in civil partnerships, where partners were aged up to 35 when they entered into civil partnership (and had come of age in the 1990s and early 2000s), Heaphy et al. report that ‘almost all the couples [they] interviewed had turned their attention to the question of becoming a parent’ (Heaphy et al., 2013: 162). However, whereas women focused on the practicalities of planning for parenthood, men’s desires were less grounded in actual plans.

Studies of sexual-minority parents who recall previous questioning of their ability to have children show that it is not uncommon for people to realise their reproductive capability at some point in their lives. Although narratives of parenting desire as ‘innate’, ‘natural’, ‘instinctive’ and relatively unaffected by one’s sexuality are not rare (Dalton and Bielby, 2000; Lewin, 1993, 2009; Murphy, 2013), there is evidence to suggest that parenting aspirations often have a contingent or situational disposition, especially among men. Using the concept of ‘turning points’, following Berkowitz and Marsiglio (2007), Jennings et al. (2014) observe that, for many lesbian mothers and gay fathers in their study, decisions to adopt were prompted by specific events and experiences. Highly significant, practically and symbolically, was the introduction of the Adoption and Children Act 2002, which allowed same-sex couples in Britain to adopt jointly. Formative experiences also included seeing lesbian and gay parents in the media, encouragement from family and friends, previous experiences with adoption, and contact with children. Studies of gay fatherhood identify ‘settling down’ and meeting gay men who are parents as other important turning points awakening men’s willingness to pursue parenthood (Goldberg et al., 2012; Lewin, 2009; Smietana, 2018). Overall, the realisation that sexual and parental identities are compatible often seems to arise through various kinds of interactions which make it possible to imagine having children outside the heterosexual context.

The sociological understanding of queer parenthood comes mainly from studies of people who already are parents and who became parents, often against the odds, in contexts that significantly differ from today’s Britain. Existing research provides less insight into the meanings of parenthood among those who have not experienced it and who are thus likely to represent a wider range of feelings about having children. In the light of still limited but growing visibility, recognition and acceptance of non-heterosexual parenthood in Britain, men and women who form same-sex relationships in the current climate may feel encouraged to entertain the possibility of having children; they may even encounter societal pressures to do so, which some may want to oppose. At the same time, cultural, structural and affective barriers may continue to obstruct the ability to imagine oneself as a parent (or to see this prospect as desirable), which is likely to vary depending on one’s position in terms of gender, class, race, age and other axes of social difference. To what extent and in what ways, then, are accounts from lesbian mothers and gay fathers reflected in views about parenthood among younger lesbians and gay men? And is there anything distinctive about how sexual minorities who had entered adulthood at a time of substantial socio-legal changes approach the possibility of becoming a parent? Although the small-scale study this article draws upon does not allow me to evaluate the extent of change or how it is perceived by different groups, it sheds light on how the cultural shift, whatever its scale and reach, manifests in people’s intimate lives and what dynamics it creates in personal relationships.

## About the study

Data presented in this article come from a qualitative interview study, which explored views about parenthood in a young generation of lesbian, gay and bisexual people in Britain. The study examined what men and women in their 20s and early 30s , who had no children, thought about becoming parents in the future. The interviews were conducted in England and Wales between 2012 and 2015.

### Background

Compared to lesbian mothers and gay fathers examined in existing literature, people in this study came of age when, legally, there were more possibilities to become parents in a non-heterosexual context. In Britain, different pathways to parenthood opened up for same-sex couples in an exceptionally short period of time. In December 2005, same-sex partners were allowed to jointly adopt (Children and Adoption Act 2002) and the rights of non-biological parents were protected through a new form of relationship recognition (Civil Partnership Act 2004). Over the following few years, it became generally easier to pursue parenthood through assisted conception. For example, the Human Fertilisation and Embryology Act 2008 facilitated access to fertility treatment for lesbian couples. Changes in the law have been accompanied by a more explicit acknowledgement of family diversity by subsequent governments and other institutions, including adoption agencies and fertility clinics, and by an increasing availability of information for prospective parents from sexual minorities.

### Participants

Interviews were conducted with 23 people, most of whom had been recruited via a dedicated study website. A link to the website was disseminated through multiple channels, including LGBT organisations, LGBT staff networks and Facebook advertisements. The website described the study as exploring what having and not having children meant to the young generation of non-heterosexual adults in Britain, and targeted people aged 20–35 who *did not* have children. Website visitors could register their interest in being interviewed by completing a short form, which asked a small number of questions, including whether the person wanted to become a parent at some point in the future. The form aimed to select a diverse group of interviewees with respect to their socio-demographic characteristics (such as ethnicity, education, employment and relationship status) as well as their views about parenthood. Owing to fieldwork constraints, only a quarter of people who had expressed interest in the study were interviewed.

Of the 23 people interviewed, 12 were men and 11 were women. Interviewees were aged between 23 and 33 years, with a median age of 28. There were 20 who identified as lesbian or gay and 3 as bisexual (none identified as transgender); 15 were in a same-sex relationship, 7 were single and one man was in a relationship with a woman. There were 19 living in England and 4 in Wales; 21 resided in urban areas and 2 in rural locations; 20 were British, 1 was American, 1 Spanish and 1 French; 19 identified as white, 2 as black, 1 as Asian and 1 as ‘other’; 17 had a university degree and 6 had completed their education at GCSE or A Levels. All but two were employed at the time of our interview and worked in a range of industries.

It should be noted that, despite the effort to recruit a diverse group of people, interviewees were predominantly urban, white and middle class. In addition, with only three interviewees identifying as bisexual, insights gained through the study largely reflect perspectives of lesbians and gay men. Data in this article in particular come from interviewees who identified as lesbian or gay. Therefore, in developing the argument presented here, and recognising the limits in its ability to apply to bisexual people, it seemed adequate to use the narrower category of ‘lesbians and gay men’.

### Interviews

I conducted 21 one-to-one interviews and one interview with a couple (all interviews were originally intended to be one-to-one but two women, who were partners, asked to be interviewed together). The interviews, all audio-recorded, lasted between one and three hours. I usually started by asking about interviewees’ initial thoughts upon finding out about the study. With each answer, I prompted them to elaborate on what they had already said. In doing so, I was guided by three broad topic areas, identified in six initial interviews (included in the final analysis): (1) thinking about parenthood (including parenting desires and intentions, or lack thereof), (2) talking about parenthood (including recollections of conversations with partners, family and friends), and (3) attitudes towards different pathways to parenthood (such as adoption, donor conception and surrogacy). This article focuses on the first two topic areas and particularly on the relationship between them, by attending to how the awareness that having children in a same-sex relationship is an option manifests in conversations and, conversely, how conversations shape people’s consciousness.

Once the interviews had been transcribed, I read each transcript multiple times, looking for themes across the interviews. My themes included pre-conceptualised thematic areas I had specifically addressed in the interviews – for example, talking about parenthood with friends – and themes identified only as I began to look for common features across the interview transcripts, such as ‘role models’. As I wrote up my analysis, I ordered the themes to form a coherent account of my interpretations of the data and, as my arguments developed, I directed my narrative towards questions provoked by ongoing scholarly debates. For analyses of data not covered in this article, see Pralat (2016, 2018, 2020).

## Findings

I present my findings in three sections before discussing them in relation to existing literature. First, I examine the presence, or relative absence, of sexual-minority parents in interviewees’ social circles and those known from the media to find that, for many people I spoke with, neither provided relatable role models, despite the fast-growing visibility of the topic of queer parenthood in popular culture. Second, I show how conversations with peers, including friends and partners, prompted thinking about parenthood, and how the idea of same-sex couples having children met with both approval and curiosity. Third, I use a case study to illuminate the specificity of contemporary same-sex intimacy in how the possibility of having children is approached in everyday life and how the awareness of this possibility not only affects couple relationships but also fundamentally alters the understanding of the relationship between reproduction, sexuality and identity. In these three data-focused sections, I refer to interviewees using pseudonyms and, when quoting, I use italics to highlight interviewees’ own emphases.

### ‘I don’t have that kind of model’

When I met Sally, a lesbian aged 31, I asked her why she had thought participating in this study was worthwhile. Pondering the possibility of having children, she said: ‘One of the things that really struck me was that we really don’t have role models for this. You know, the concept of the family has changed *so much* over the last … throughout our lifetime. And I didn’t really know … how to deal with that.’ Louis, a 24-year-old gay man, echoed Sally’s sentiments: ‘I don’t know anyone, I don’t have any friends or anyone in my social environment who is in a gay couple and has children. I have many gay friends, but I don’t have *that* kind of model.’ The lack of direct points of reference to queer parenthood clearly affected Louis’ views about having children, which remained ambivalent: ‘Maybe if I actually knew people and I knew that they were *happy* like that – they had been through that process and come out happy – it would be different.’

The absence of role models seemed more problematic to interviewees who were undecided about their own feelings regarding parenthood. For people, especially women, who were certain about their parenting desires, proficient at navigating available resources, and supported by partners, family and friends, knowing sexual-minority parents at the stage of planning for parenthood, while preferable, was not necessary. Katie, a lesbian aged 31, who was hoping to become pregnant via home insemination with her partner, commented:

We don’t know any other lesbian couples that have children or that are wanting to have children. And we do see this as a problem. We do want some peers that we can share this experience with. So we’ve been looking for support groups and we know of a few. We know where they meet, but it doesn’t quite feel appropriate for us to attend, at least until we’re pregnant. But it’s nice to know they’re there.

Although Katie and her partner did not feel comfortable seeking contact with lesbian parents until they were already in the process of transition to parenthood, they felt confident enough to start a family without making such contact. In this case, knowing lesbian mothers was not important for proving that the couple could have children, or that they could be good or happy parents. Rather, the women contended that having access to people with a ‘similar family makeup’ would make them feel less unusual and, most crucially, it would protect their future children from feeling different.

Generally, knowing queer parents was not common among the 23 people I spoke with. Only one interviewee had non-heterosexual parents among her close friends; 11 could identify such parents among people they knew or knew of. Apart from one interviewee who said that he knew gay fathers – specifically, men who had become parents in previous heterosexual relationships – everyone else referred to women, half of whom had come out as lesbians later in life, with children from prior straight unions. Although such lesbian mothers were the most common queer-parent figures, they were often seen as a separate category, implicitly ascribed to earlier generations. Lesbians who had children *after* coming out included women who had got pregnant via ‘home AI [artificial insemination]’, who had ‘met a guy on the internet and had a baby with him and his boyfriend’, or who had ‘gone out, found a mate, mated and had a baby’. There were no recalled cases of sexual-minority parents who had adopted, fostered, pursued surrogacy or used a fertility clinic.

Queer parents known from the media were a gender reverse of those known from the ‘real world’. When I asked about famous people, no interviewees mentioned well-known lesbian mothers, but nine were able to name prominent gay fathers. However, the public image of gay fatherhood was usually reduced to one person: Elton John. Seven interviewees, most of whom could not point to anyone else, referred to the British singer who had two sons via surrogacy. Although some acknowledged Elton John’s role in normalising non-heterosexual family life, the singer’s wealth, celebrity status and older age overshadowed his sexual identity and neither men nor women could relate to him as a role model.

While there was an invariably recognised dearth of familiar sexual-minority parents in popular culture, interviewees seemed to agree that there had been a fast-growing visibility of the *topic* of queer parenthood in the media. Many were able to recall reading articles or watching TV shows that focused on the subject. It was not only interviewees’ awareness that had been shaped by media encounters, but also their interactions with others. Becky, a 25-year-old lesbian who wanted to have children with her partner, told me that reading an article about ‘lesbian insemination’ had made her ‘think a lot about what we will be doing’. The article was especially important as it had been emailed to Becky by her mother: ‘I took that as a *massive* vote of confidence from mum, given that she’s not really vocalised [her views] very much. So *that* was kind of a breakthrough.’ Louis admitted that television had played an important role in increasing his awareness of sexual-minority parenthood. Referring to American drama series *Six Feet Under* and *The L Word*, he said that storylines about queer people becoming parents had made him appreciate what creating a family might involve in his circumstances: ‘It’s not like “this is easy”. It is complicated but it doesn’t mean that it’s bad.’

Louis understood his experience as generationally specific, marked by a rapid shift in what has been culturally available for imagining one’s intimate future as a non-heterosexual person: ‘I just suspect that it would have been very different for someone who is maybe five years older. And also that it’s very different for people who are teenagers now.’ He reflected on his early adolescence when, upon realising that he was gay, he started to ‘panic’ because he ‘didn’t know what was meant to happen over the course of my life’: ‘I think, you know, there were representations available in the media, it wasn’t like there was nothing. But you kind of had to find them. Because there was zero conversation about this happening around you.’ Nathan, a gay man aged 26, made a similar observation when he recalled growing up: ‘Thinking back to my childhood, the very idea of a gay couple adopting, you wouldn’t have seen that on television or in the papers.’ Nathan, like Louis, acknowledged the speed of social change with respect to media representations of queer-parent families, situating his life as encompassing a sizeable amount of this cultural transition, but he also highlighted that the idea of non-heterosexual parenthood was still culturally new: ‘It’s not quite a non-issue, but it’s not seen as a taboo subject, it’s not a scandalous subject. It’s still not utterly mainstream – it is still a newsworthy story.’

The ‘newsworthiness’ of queer parenthood meant that, despite the relative absence of actual sexual-minority parents in interviewees’ lives, the awareness of parenthood as a possibility was high. But, as I show in the next section, while it had clearly become more *accepted* for same-sex couples to have children, whether it was *expected* of them to do so remained uncertain.

### The curious case of queer parenthood

Echoing a sentiment expressed in a number of interviews, Amit, a gay man aged 31, reflected on what people in his social circles thought about having children: ‘Very few of the straight couples I know want to remain childless – I think there’s one or two who have kind of taken that decision. But for the non-straights, I really couldn’t say.’ Amit’s uncertainty about parenting desires of other queer people in his peer group made him unable to evaluate whether his own views about having children fit or departed from the norm – or whether there was a norm at all. Maria, a 31-year-old lesbian, observed towards the end of our interview: ‘You’ve probably made me more curious now to actually, maybe even ask some of my friends more directly, like, about plans – now I’m interested to know if some of my gay friends feel similarly [about] parenthood.’ In general, the men and women I spoke with seemed to have limited ‘inside knowledge’ about how widespread or uncommon it was for sexual-minority people to want to have children. They also seemed unsure whether the ‘default future’ for same-sex couples in today’s Britain was child-centred or childfree.

Crucially, the uncertainty about what interviewees’ non-heterosexual contemporaries thought about having children contrasted with seemingly clearer perceptions of both previous generations of sexual minorities and of heterosexual peers. In the past, as discussed earlier in the article, being a lesbian mother or a gay father ‘deviated’ from the presumed norm of lesbians and gay men as childless. These days, my interviewees suggested, though it seemed increasingly accepted to be childfree, being a straight non-parent was still at odds with the cultural expectations of the mainstream society. Situated in relation to queer predecessors and heterosexual coevals, both with apparently more clearly defined norms, interviewees occupied a temporal space where their relationship to reproduction seemed more ambiguous.

What was clear from my interviews was that, in most cases, parenthood only became relevant in the context of a long-term relationship. Being part of a ‘stable’, monogamous couple was seen as a prerequisite for more concrete considerations about becoming a parent – both by interviewees who were partnered and by those who were single. People I spoke with often made distinctions between thinking about parenthood in ‘hypothetical’, ‘theoretical’ or ‘abstract’ terms and approaching the topic ‘more seriously’, with the latter happening almost exclusively in couple scenarios. A serious dealing with the subject required an understanding of whether two people had compatible ideas about their intimate futures.

Sometimes one partner’s parenting desire was so evident that no ‘clarification’ of feelings about parenthood was needed. ‘When you meet [my partner], you realise that it’s just part of [her] – she’s just always wanted to have children,’ said Vicky, a 28-year-old lesbian in a civil partnership. ‘So it’s never been brought up in that kind of way.’ For Vicky, whose ideas about the future had initially differed from her partner’s, the potential incompatibility necessitated an internal reflection rather than an open negotiation:

When I met my partner, I knew that I *didn’t want* to have children and she *very strongly did*. And in the very early stages of the relationship, when you’re kind of getting to know each other, I realised that I had to be okay with the idea of kids, ‘cause it’s not something she’s going to budge on.

In most relationships that interviewees told me about, the issue of having children or remaining childfree was not as unequivocal as in Vicky’s case. Especially among men, partners remained largely unsure about each other’s views for some time. Gavin, a gay man aged 25, could not recall talking about parenthood with his partner in the first seven years of their nine-year-long relationship. His first recollection of having ‘this kind of conversation’ was when the couple’s close friend, a heterosexual woman, was expecting her child:

Once she was pregnant, she said something like, ‘Oh, would you like to have them?’ And we both just kind of looked at each other and I was like, ‘Oh, I think I’d like to.’ And [my partner] said, ‘I think I would.’ So I think it wasn’t either of us that really had the idea – or if we did, we didn’t say anything. I think it was [my friend] who instigated it.

Gavin described having the memorable conversation as a moment when ‘the seed was sown’, before observing that, more recently, he had been asked about his family plans increasingly often: ‘I think people are curious more than … They’re not asking genuinely, they’re just curious.’ When I asked him what he thought people were curious about, he said: ‘Well, I don’t know, I suppose the whole process as opposed to if we’re planning to have a child. But I’m always kind of suspicious, I think, when people do that, because … you know, I always think, “What are you trying to get out of there?”’

Some interviewees mentioned that their heterosexual peers had few gay people among their friends and, aware of the emerging possibilities for same-sex couples, wondered if parenthood was on the agenda for the non-heterosexuals they knew. Becky commented:

My boss is always *mega*-interested. Not in a pervy way! [*laughs*] But he just doesn’t have anyone in his social circle who’s gay. He’s got two kids on his own, so he sort of explains how it was from his point of view, and then sort of says, ‘Well, how do you guys do it?’

While the process of becoming a parent as a same-sex couple seemed to arouse most curiosity, the question of whether having children was a plan was also common and it was usually directed at women. Some interviewees argued that it was inappropriate to ask others about their parenting intentions, yet it seemed ‘socially acceptable’ to do so. Sally, in a same-sex relationship for six years and still undecided whether she wanted to have children, had been asked about her childbearing plans by ‘literally everyone’: ‘I just say no. It kind of ends the conversation. [*laughs*] That, or I say, “No, I have three cats.”’ Sally recognised that the question was insensitive but, when directed at same-sex couples, it was often a well-intended acknowledgement of their ability to become parents: ‘You know, in some ways it’s great. ‘Cause previously they would’ve thought, “She’s gay, clearly not gonna happen.” So, I mean, it’s fantastic that people are all thinking that is a reality for me. But at the same time it is slightly annoying.’

At times, the question about parenting intention led to situations that were rather uncomfortable. Lauren, a 30-year-old lesbian who was single and uninterested in having children, recalled being asked ‘Are you planning to have kids?’ when she and her ex-girlfriend were in the process of trying to reconcile their divergent attitudes towards parenthood – a decisive factor in their subsequent breakup: ‘They were asking us when we were both in the room and I was just going, “Um, I don’t know. I don’t really want to talk about that.” And I said to my friend [later], “That was the worst thing you could have brought up!”’

In the next section, I use Lauren’s story as a case study to demonstrate in greater detail why questions about parenthood can be difficult to answer, even when there is seemingly little ambivalence in how one feels about having children.

### ‘This is a conversation I will actually need to have’

Lauren’s view about parenthood differed from that of her former partner. While still in the relationship, she pondered ways in which the couple could address their disparate feelings about having children to stay together, but concluded that it was a no-compromise issue: ‘It’s not like you can have kids a few days a week or you can say, “Oh well, you don’t like that, I like that, let’s meet in the middle.” It’s either a kid or no kid, isn’t it?’ Faced with a conundrum in her relationship, Lauren re-evaluated her understanding of what it meant to be a lesbian. In our interview, she reflected on her perceptions at length, going back to the time before meeting her ex-girlfriend:

I had always presumed – and I think it was naïve, it was up until my kind of mid-20s – that most lesbians wouldn’t want kids. And I think I just presumed that everyone was *not* gonna adhere to that heteronormative lifestyle and that, you know, it was a rare thing that lesbians would decide to go and have babies. So it was quite a shock when I realised that actually some, quite a lot of lesbians want kids. And it was something that was actually gonna have to be a conversation. I remember I had two dates with two different people and on the first date they asked me if I wanted kids. And I remember just being like, ‘What the fuck?! That’s a *serious* conversation for a first date!’ [*laughs*]

In her teens and early 20s, Lauren thought that coming out as a lesbian automatically rendered parenthood irrelevant. Progressing through her adulthood, she realised that, in fact, many lesbians did want to have children, even if it seemed like a ‘heteronormative lifestyle’. Increasingly aware of her own lack of desire to become a parent, in what could be described as a consciousness-raising moment, Lauren identified the issue of parenthood as ‘something that was actually gonna have to be a conversation’.

During the three-year-long relationship with her ex-partner, Lauren understood that parenting intentions among lesbians were not uncommon, which reflected, in her view, both the fact that her peers had been reaching their 30s and the social changes in the arena of queer parenthood. A few months after breaking up with her girlfriend, and shortly before our interview, Lauren felt ready to start dating again, although she was not yet prepared for another relationship. However, setting up an online dating profile brought about an unforeseen dilemma:

You know, [the online dating website I’m using] asks about your height, your star sign, your marital status, whether you’re this, whether you’re that, and [then it asks], ‘do you want children?’ And I looked at it and I was like, ‘Oh god, I don’t know what to put here!’ Because if I put ‘no’, probably a lot of people would never even talk to me. *But* I’m not looking for a relationship on here at the moment. I want some *dates* and I want some *fun* and I want a laugh and I want to meet some women and, you know, have a bit of a fling. *So* I had this proper turmoil. I eventually put ‘undecided’ on this thing. But I’m *also* aware that, previous to us getting together, my ex had used this website. And at *some* point – it’s a *small* pool in [our city] – she’s gonna join it again, and she’s gonna pop up. And it’ll *break my heart* when she pops up and I realise that she’s joined it. And I have this proper guilt thing – ‘cause she’s gonna see it and it’s gonna say, ‘Do you want children? Undecided.’ And she’s gonna look at it [and think], ‘“Undecided?” Un-fucking-decided? Has she changed her mind?!’

Even though it was clear for Lauren that she did not want to have children, expressing her view about parenthood on an online dating profile was no easy matter. Being explicit about her lack of parenting desire while seeking potential lovers was likely to reduce an already scant number of women ‘available’ to date. The small size of the local lesbian community, mirrored in its virtual infrastructure, had other implications too. Lauren’s ex-girlfriend was likely to encounter what would probably be a confusing piece of information that would call into question the official reason why the couple had parted ways. What initially might have seemed like a straightforward box-ticking exercise left Lauren torn between wanting to avoid the risk of ‘heart break’ and the willingness to maximise her dating success by obscuring her feelings about parenthood.

Lauren’s former partner aside, the question about having children was still difficult to answer since even responding ‘undecided’ only postponed what ultimately needed to be a binary choice. Towards the end of our interview, Lauren observed: ‘This is an issue that is going to keep coming up. When I’m *ready* for a relationship, when I am actually properly looking around and ready to meet someone *serious*, this is a conversation I will actually need to have.’

Lauren’s story captures various aspects of the cultural shift this article aims to explain. Her anecdotes illustrate that the issue of having children is relevant not only to those lesbians and gay men who want to become parents – to varying degrees, it concerns anyone engaging in romantic encounters. Based on Lauren’s account – which is consistent with my other interviews – in previous generations, adopting a lesbian or gay identity was likely to preclude reproductive practices. This has changed, or is changing, and while the change opens up new possibilities for sexual minorities by expanding the notion of the family, it also destabilises what might have been taken for granted in the not-so-distant past – that entering into a same-sex relationship means no kids in the future. Recognising that the question of whether or not someone wants to have children is relevant to same-sex intimacy, and that it often needs to be explicitly answered, reveals that parenting desire is no less significant than sexual desire in determining what kind of relationships people enter into – or decide to continue or abandon. As Lauren’s case illuminates, people’s perceptions of themselves and of their intimate lives can be shaped just as much, if not more, by a desire to have a child or to remain childfree as they are by a desire for a romantic partner.

Lauren ended up opting for ‘undecided’ on her online dating profile but, based on her prior dating experience, it was likely that she would at some point be asked for a clarification of her feelings about parenthood. Sooner or later, she would need to ‘come out’ as *not wanting to have children*. I am using the metaphor of coming out in this context to highlight a parallel between sexuality and reproduction in how desires, attractions and yearnings are organised to form identities or ‘orientations’. By bringing together my findings and existing scholarship, I will now develop this argument further.

## Discussion

Drawing on interviews with lesbians and gay men who do not have children but may have them in the future, I have explored how people who form same-sex relationships think and talk about parenthood. We have seen that, despite notable cultural changes, existing queer parents are often absent in people’s lives. The media and wider social circles provide ‘anecdotal evidence’ of lesbian-mother or gay-father families, but there is a shortage of relatable role models. Based on my interview data, it can be unclear to non-heterosexual people whether, in the light of the new socio-legal opportunities, they are socially expected to become parents and what their queer peers are more likely to want. At the same time, straight people, voicing their approval, are often curious if their lesbian and gay friends plan to have children. The ambiguity about parenting intentions may lead to some ‘awkward encounters’ – between gays and straights but also among non-heterosexuals themselves. Queer parenthood may have become more thinkable and ‘talkable’, but the ideas that move between people’s consciousness and their conversations have no established paths to follow.

Berkowitz, in her study of gay fatherhood in the USA, argues that younger men ‘have visible openly gay fathers as models who in their everyday actions are transforming what it means to be a gay man’ (Berkowitz, 2007: 179). My data provide no evidence of significant presence of ‘role models’, let alone their transformative influence, in the lives of lesbians and gay men in Britain. Most interviewees in my study did not know any queer parents and found it difficult to relate to those visible in the media (usually, gay fathers via surrogacy) due to their celebrity status, wealth and older age. The perceived absence of existing families serving as models – in the sense of offering advice, inspiration or encouragement – echoes retrospective accounts of lesbian mothers and gay fathers from previous generations (Gianino, 2008; Hequembourg, 2004; Touroni and Coyle, 2002). Those parents, sometimes described as ‘pioneers’ (Dunne, 2000; Ryan-Flood, 2005; Stacey, 2006), often point out this absence among the challenges they faced when creating their own families. My findings suggest that the next generation of sexual-minority parents is likely to have their own pioneering experience. This is not to say that lesbians and gay men in Britain do not perceive increasing possibilities of creating families outside the heterosexual realm – even if these are largely limited to couple relationships (Gabb, 2018; Pralat, 2018; Reed, 2018). However, this awareness seems to be facilitated not so much by tangible families providing exemplars one could follow or aspire to, but rather by more general shifts in public consciousness where the equation of homosexuality with childlessness gradually disappears.

Similar to research on lesbian mothers and gay fathers (Berkowitz and Marsiglio, 2007; Jennings et al., 2014), notable in the narratives of people I spoke with were specific ‘turning points’ – events and encounters that had made them more aware of their ability to become parents. In particular, casual conversations with friends (usually, straight peers) played an important role in expanding this awareness, as they provoked more intimate conversations between partners – at times accentuating a compatibility of views about parenthood, at other times highlighting that the views were incompatible. The concept of turning points is useful for understanding the dynamic relationship between sexuality and reproduction, and how it figures in identity formation. As Ahmed (2006) observes, life is full of turning points and, depending on which way one turns, different worlds might come into view. In her analysis of the concept of ‘sexual orientation’, and the idea of being ‘orientated’, she notes that ‘orientations toward sexual objects affect other things that we do, such that different orientations, different ways of directing one’s desires, means inhabiting different worlds’ (Ahmed, 2006: 68). The very existence of lesbian mothers and gay fathers evidences that deviating from the ‘straight line’ does not require ‘turning away’ from reproduction. But, as both my findings and studies of queer parents demonstrate, aligning same-sex desire with a desire to have a child can have disorientating effects.

The feeling of disorientation is likely to result from a lack of clarity about one’s life trajectory or, as Louis put it, not knowing what is ‘meant to happen over the course of my life’. This is consistent with existing literature. For example, in their study of civil partnerships, Heaphy et al. observe that, in the context of changing conventions, it can be more difficult for same-sex couples ‘to move on to what might be regarded as the “next stage” of a relationship’ (Heaphy et al., 2013: 165). As my data illuminate, the possibility of parenthood is an important aspect of this ambiguity and, in developing a romantic relationship, it is potentially challenging to find a good time when partners’ visions of their intimate futures can be mutually clarified. It seems that, in Britain, talking about having children in a non-heterosexual context is still culturally new. Despite the growing visibility, acceptance and recognition, queer parenthood remains to a large extent ‘unscripted’ (Hequembourg, 2004) – there is a dearth of cultural scripts to follow or social guidelines to adhere to (Almack, 2005).^2^ The absence of established narratives means that becoming a parent can feel like entering ‘uncharted territories’ (Nordqvist and Smart, 2014). Conventions about raising the topic in different situations, and the implications it has for intimate life, are in flux – and they are not necessarily more obvious to people who form same-sex relationships than they are to their inquisitive heterosexual peers.

This lack of clarity necessitates moments of clarification. I argue that, whatever their feelings about parenthood, many queer people in contemporary Britain have to ‘come out’ as wanting, or not wanting, to have children. Similar to sexual identity, in some cases, a particular ‘reproductive orientation’ might be so evident that coming out is not necessary. Likewise, feelings about parenthood can remain ‘in the closet’ for some time, go through periods of ‘questioning’ or, indeed, be fluid and change over time through various kinds of encounters. Crucially, analogous to sexuality, when understood as a framework for identity formation, reproduction seems to be governed by binary logic, making it difficult to occupy a ‘middle ground’. Just like there is space for bisexuality, there is room for fostering and other adult–child relationships that do not fall easily into the categories of ‘parent’ and ‘non-parent’. But these middle positions, while not uncommon, are relatively invisible and marginalised (Dempsey, 2012; Moore, 2011; Reed, 2018).^3^ As such, they can also be more difficult to imagine. ‘It’s either a kid or no kid,’ Lauren observed, as she described how she and her partner had been unable to ‘meet in the middle’.

However, using the concept of coming out when drawing an analogy between sexuality and reproduction reveals not only the persistence of binary thinking but also a potential opportunity. The current cultural moment, when meanings of ‘the family’ and of what it means to be lesbian or gay expand, suggests that it is possible for parenthood and ‘childfreedom’ to relate to one another in a neutral way, with neither favoured or privileged over the other – and with neither explicitly normative. Data presented in this article show that, on a personal level, the ambiguity surrounding cultural expectations about having children can feel confusing. It is easy to envisage a gradual ‘transition’ towards clearer norms where, eventually, in an increasing number of contexts, same-sex couples will be as socially expected to become parents as their heterosexual counterparts. But it is also possible to imagine the uncertainty surrounding parenthood to persist – and people getting used to it or even taking it for granted. If this ambiguity in queer kinship is more permanent than temporary, it can establish parenthood and childfreedom as intimacies of equal value. Perhaps, at some point in the future, this normative challenge will expand to the heterosexual mainstream. And maybe, some day, coming out in its original ‘sexual’ sense will concern not a ‘disclosure’ of non-normative identities but a general expression of romantic leanings.

## Notes

1 I use the term ‘queer parenthood’ as a broad category that encompasses lesbian motherhood, gay fatherhood, bisexual parenthood and other forms of non-heterosexual or sexual-minority parenthood.

2 This is in contrast to ‘highly scripted’ gendered conventions of pursuing parenthood in a heterosexual context, as shown, for example, in research on infertility treatment and assisted reproductive technologies (Franklin, 2013: 234; Thompson, 2005: 118–119).

3 I am aware that my lack of success at recruiting more bisexual interviewees, and the resulting focus of this article on lesbians and gay men, unfortunately contributes to the invisibility and marginalisation of bisexuality.
